# State dependence explains individual variation in nest defence behaviour in a long‐lived bird

**DOI:** 10.1111/1365-2656.13411

**Published:** 2021-01-08

**Authors:** Margje E. de Jong, Marion Nicolaus, Rienk W. Fokkema, Maarten J. J. E. Loonen

**Affiliations:** ^1^ Department of Behavioral and Cognitive Biology University of Vienna Vienna Austria; ^2^ Arctic Centre University of Groningen Groningen The Netherlands; ^3^ Conservation Ecology Group Groningen Institute for Evolutionary Life Science (GELIFES) University of Groningen Groningen The Netherlands

**Keywords:** behavioural reaction norms, boldness, nest defence, personality, phenotypic plasticity, risk‐taking, selection, senescence

## Abstract

1. Parental care, such as nest or offspring defence, is crucial for offspring survival in many species. Yet, despite its obvious fitness benefits, the level of defence can consistently vary between individuals of the same species. One prominent adaptive explanation for consistent individual differences in behaviours involves state dependency: relatively stable differences in individual state should lead to the emergence of repeatable behavioural variation whereas changes in state should lead to a readjustment of behaviour. Therefore, empirical testing of adaptive state dependence requires longitudinal data where behaviour and state of individuals of the same population are repeatedly measured.
2. Here, we test if variation in states predicts nest defence behaviour (a ‘risky’ behaviour) in a long‐lived species, the barnacle goose *Branta leucopsis*. Adaptive models have predicted that an individual's residual reproductive value or ‘asset’ is an important state variable underlying variation in risk‐taking behaviour. Hence, we investigate how nest defence varies as a function of time of the season and individual age, two state variables that can vary between and within individuals and determine asset.
3. Repeated measures of nest defence towards a human intruder (flight initiation distance or FID) of females of known age were collected during 15 breeding seasons. Increasing values of FID represent increasing shyness.
4. We found that females strongly and consistently differed in FID within‐ and between‐years. As predicted by theory, females adjusted their behaviour to state by decreasing their FID with season and age. Decomposing these population patterns into within‐ and between‐individual effects showed that the state‐dependent change in FID was driven by individual plasticity in FID and that bolder females were more plastic than shyer females.
5. This study shows that nest defence behaviour differs consistently among individuals and is adjusted to individual state in a direction predicted by adaptive personality theory.

## Graphical abstract

In line with adaptive personality theory, this study shows that nest defence differs consistently among barnacle goose females and is adjusted to individual state (season and age). Additionally, this study revealed that behavioural types differ in their level of plasticity, with bolder females being generally more plastic than shyer females.

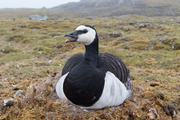

## 1 INTRODUCTION

Parental care is known to be crucial for both offspring and parental fitness (Clutton‐Brock, 1991; Klug & Bonsall, 2014; Royle et al., 2012). Yet, despite its obvious fitness benefits, there is huge variation in this behaviour among species, ranging from limited short term to extensive long‐term parental care (Royle et al., 2012). Intriguingly, the amount of parental care can also vary among individuals of the same species. For example, many studies have reported, for a wide range of taxa, that individuals or pair members differ consistently in their nest or offspring defence behaviour over time or across contexts (e.g. mammals: Bubac et al., 2018, fish: Stein & Bell, 2015, birds: Burtka & Grindstaff, 2013; Clermont et al., 2019; Kontiainen et al., 2009; Patrick et al., 2013; Thys et al., 2019). Such consistent individual differences in behaviour (also referred to as ‘animal personality’) are intriguing because it implies that individual parents express limited plasticity in offspring care; that is, the range of plasticity an individual can display is smaller than the behavioural diversity that exists in the entire population (Sih et al., 2004). This raises the question why variation in offspring care arises and is maintained within populations.

Parental care in the form of nest and offspring defence directed against predators can be viewed as a ‘risky’ behaviour as it involves benefits by increasing offspring survival, but also comes with the risk of injury and possibly even death for the parents themselves when they, for example, attack or try to distract a predator (Alsonso‐Alvarez & Velando, 2012; Montgomerie & Weatherhead, 1988). Most adaptive explanations for consistent individual differences in ‘risky’ behaviour involve differences in stable state (Dingemanse & Wolf, 2010; Wolf & Weissing, 2010). State of an animal represents ‘*all those features that are strategically relevant, i.e. features that should be taken into consideration in the behavioural decisions in order to increase fitness*’ (Wolf & Weissing, 2010). Consequently, when ‘state’ changes, individuals should adjust their behaviour accordingly (state‐dependent behaviour, phenotypic plasticity; e.g. Wolf & Weissing, 2010). For example, one widely recognized state variable that can give rise to consistent individual differences in risky behaviours is an individual's residual reproductive value (RRV) or ‘asset’ (Wolf et al., 2007a, 2007b). The ‘asset protection’ principle states that individuals with a high asset, that is, high RRV, should behave risk‐averse in order live long enough to harvest this asset, while individuals with a low asset, that is, low RRV, should take more risks as they have less future fitness to lose (Clark, 1994). Theoretical studies have confirmed that if there are stable differences in individual asset and/or there is a positive feedback between asset and behaviour, individuals exhibit long‐lasting consistent differences in risky behaviour (Wolf et al., 2007a, 2007b; Wolf & Weissing, 2010). However, if individual asset is changing, adaptive plasticity of the trait is expected (Nicolaus et al., 2012).

Empirically, studies across taxa report evidence for relationships between asset and risk‐taking behaviour that are in line with theory (e.g. bird: Hall et al., 2015; mammal: Dammhahn, 2012; insect: Moschilla et al., 2018; crustacean: Ory et al., 2015). Specifically, differences in offspring defence behaviours of parents have been attributed to variation in state variables such as breeding experience, age, body condition, reproductive stage and timing, reproductive value and food abundance (e.g. Bubac et al., 2018; Clermont et al., 2019; Kontiainen et al., 2009; Seltmann et al., 2012; Thys et al., 2019). Yet, to disentangle whether such behavioural variation originates from within‐ or between‐individual effects in state, we need sophisticated statistical methods that are notoriously data hungry and require large datasets with many individual repeats (Brommer, 2013; van de Pol, 2012). Such longitudinal datasets are not easily obtained in the wild and can be insufficient for short lived species. Longitudinal studies on long‐lived animals are ideally suited to fill this gap.

**FIGURE 1:**
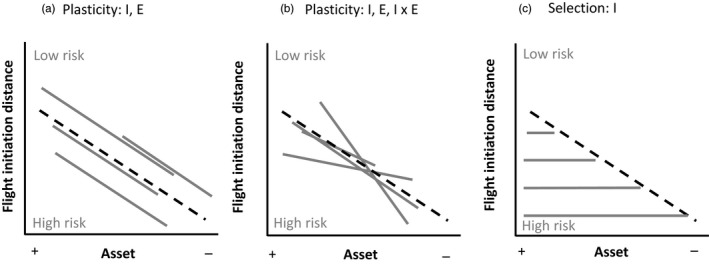
Examples of three behavioural reaction norms (BRN) that depict a decline in population mean flight initiation distance (FID, dashed black line) with decreasing asset but involve different types of individual responses (grey lines): (a) Population decline is caused by state dependence of FID in absence of individual variation in plasticity. Females differ in asset and mean FID (elevations of BRN) and reduce FID with decreasing asset in a similar way (slopes of BRN; I, E). (b) Population decline is caused by state dependence of FID in presence of individual variation in plasticity (I, E and I × E). (c) Population decline is caused by selection, for example, the selective disappearance of shy individuals (between‐individual effect; I, no E)

This study aims to test whether variation in individual ‘state’ predicts nest defence behaviour in free‐living female barnacle geese *Branta leucopsis*. For 15 years, repeated measures of female nest defence towards a human intruder (flight initiation distance or FID) were collected during the breeding season. Increasing values of FID represent increasing cautiousness (also called shyness: Blumstein et al., 2016; Réale et al., 2007). We predict that FID should vary as a function of time of the season and individual age, two state variables that can vary within and between females and can determine asset: (a) net benefits of parental investment in nest defence may increase over a season through a decrease in parental renesting ability (Barash, 1975; Curio et al., 1984) and/or through an increase in current offspring value, as the replacements costs of these offspring increase when they get older (parental investment theory: Trivers, 1972; but see Dawkins & Carlisle, 1976; Boucher, 1977; for review see Montgomerie & Weatherhead, 1988; Caro, 2005), and (b) older individuals that are less likely to survive and reproduce in the future are predicted to take more risk to defend their current offspring than younger individuals with higher future fitness expectations (e.g. Class & Brommer, 2016). To test these predictions we used a behavioural reaction norm approach to study individual variation in plasticity and mean ‘risky’ behaviour (individual slope and elevation of the reaction norms respectively) over an environmental gradient (change in state; de Pol & Wright, 2009; van Dingemanse et al., 2010). We generally hypothesize that population mean FID should decrease with time of the season or population mean age but highlight three non‐exclusive scenarios that can explain this pattern: (a) The pattern is explained by *behavioural plasticity* (within‐individual effect). In this case, females differ in asset and mean FID (differences in elevation) but reduce FID with season or age either in a similar (individual slopes are similar; Figure 1a) or dissimilar manner (presence of individual by environment interaction I × E, individual slopes are dissimilar; Nussey et al., 2007; Figure 1b). (b) The pattern is caused by *selection*, that is, the selective (dis)appearance of certain individuals; here for example the selective disappearance of shy individuals (between‐individual effect; Figure 1c). In this case, females differ in asset and mean FID, but do not exhibit plastic behavioural change (the slopes are null). Within a breeding season, this could indicate brood failure of shy individuals, while over age this could indicate that shy individuals suffer higher mortality. (c) The pattern is caused by a *combination of plasticity and selection* (not shown in Figure 1).

## 2 MATERIALS AND METHODS

### 2.1 Study system

We performed this study using a 15‐year dataset (2001, 2003, 2005–2017) of a barnacle goose population nesting on the islets Storholmen (*c*. 30 ha) and Prins Heinrichøya (*c*. 3 ha) in Kongsfjorden, near the village of Ny‐Ålesund (78°55′N, 11°56′E), Spitsbergen (Svalbard). The geese arrive at the breeding ground late May/early June from their wintering area in the United Kingdom. Barnacle geese have a high breeding site fidelity (Black, 1998). Geese usually start nesting a few days after arrival, although some pairs postpone laying for up to 2 weeks (Dalhaug et al., 1996). The period between laying and hatch takes approximately 29 days, and goslings fledge at about 6 weeks of age (Lameris et al., 2019; Owen & Black, 1989). Recently, with increasing spring temperatures, Kongsfjorden barnacle geese have advanced their timing of reproduction (Lameris et al., 2019).

### 2.2 General goose monitoring; sex and age

Nest monitoring took place each year approximately every other day during the incubation and hatching period from June to the beginning of July. Geese were identified by individually recognizable engraved plastic leg rings (see below) when the researcher approached the nest and sex was attributed to them on the basis of these observations. Sometimes, however, individuals were attributed different sexes in subsequent years. Of 1,134 females and males in the database, 479 individuals were sighted ≥three times and had the same sex attributed to them every year or in ≥75% of the sightings. For these individuals we considered the sex that was attributed to them during nest monitoring as plausible. For individuals that were sighted less often or had a different sex attributed to them during different sightings, we used data from moult catches as follows. Mass captures of moulting goose flocks happened at the end of July/beginning of August (Loonen et al., 1997). During these catches, geese were ringed with individually recognizable engraved plastic leg rings on one leg and metal rings on the other and were sexed by cloacal inspection. There was a very strong correlation between the sex of the individuals as assigned to them at the nest and the sex that was attributed to them during catch (*N* = 445, Kendall's rank correlation tau: 0.95, *z* = 20.12, *p* < 0.001). Therefore, we assigned the sex observed during catch to individuals that were sighted less than three times or of which less than 75% of the sightings attributed to them were of the same sex.

Age was assigned to an individual during moult catch. Geese that were caught as goslings could be assigned an exact age (age is 0 in year of first catch), while we assumed that other geese that were caught for the first time as adults were 2 years old, as this is the minimum age barnacle geese are physically capable of reproduction (Prop et al., 1980). The median age of female geese in this dataset was 7 years (*N* = 448).

### 2.3 Observations on flight initiation distance

During incubation, multiple observations of nest defence of individually identifiable females against a threatening stimulus (human observer) were conducted every season by assessing FID. FID is defined as the number of paces between the observer and the female at the moment the female goose flees of her nest, during a straight and slow walking approach of the observer towards the nest (see for similar methodology; e.g. Miller et al., 2013; Osiejuk & Kuczyński, 2007; Quillfeldt et al., 2005; Sjöberg, 1994). Most females walked or ran off their nest, only in some cases (usually when they bred on the edge of an island) they flew off and landed in the water. FID measurements were not randomized in space and time as they were collected during standard nest checks, but the route past the nests changed between visits to the colony. We assume that a small FID equals high nest defence and high risk‐taking, and the opposite for a high FID (Blumstein et al., 2016). Observations of FID within a year were not taken into account when these observations were made (a) after a female goose was caught on the nest or (b) when eggs were collected for other research purposes, as these activities might have affected further measurements. Females that fled their nests before the observer could make his/her approach were not used in the analyses. Over all study years, 4353 successful FID observations of 465 females were done, with on average 290 observations per year, by nine different observers.

### 2.4 Statistical analyses

We analysed the data using R version 3.5.1 (R Core Team, 2018). For all analyses of the FID data, we applied a square root transformation to normalize the data.

#### 2.4.1 Repeatability

We analysed the repeatability of female FID at different temporal scales to assess the consistency of this behaviour, using linear mixed‐effects models (LMM; R package lme4: Bates et al., 2015) with a random effects structure as proposed by Araya‐Ajoy et al. (2015). We fitted a model with two random effects: individual identity (ID) and the combination of ID and year (‘ID‐year’). To control for the possible confounding effect of observer, we fitted observer as a fixed effect, which enabled the calculation of ‘adjusted repeatability’ (Nakagawa & Schielzeth, 2010). Using the variance values from this model, we assessed the repeatability of female FID on the long term (i.e. between‐individual) versus short‐term (i.e. within‐individual‐between‐year). Long‐term repeatability was calculated by dividing the variance of ID (i.e. variance between‐individuals) by the sum of variance components ID, ID‐year (i.e. variance within‐individuals/between‐years) and residual variance (i.e. variance within‐individuals/within‐years). Short‐term repeatability was calculated by dividing the sum of ID and ID‐year by the sum of variance component ID, ID‐year and residual variance (Araya‐Ajoy et al., 2015; Clermont et al., 2019).

#### 2.4.2 Testing state dependence of FID

To test whether nest defence behaviour increased with reduced asset, that is, with date within year or age across years, we conducted two sets of analyses quantifying how FID varied in relation to either date or age (model #1).

First, we analysed variation in FID as a function of date by fitting centred June day (from now on only ‘June day’) on which FID was measured as a fixed effect. June day was mean‐centred for each year to correct for annual variation when the FID measurements were done: we subtracted the mean June day of each year from each June day on which a FID measurement was taken of an individual during this particular year. We used June day to model the seasonal gradient as opposed to nest stage (which is used in comparable studies e.g. Clermont et al., 2019; Thys et al., 2019), because this allowed us to take the whole dataset into account as we only have hatch dates for successful nests and lay dates were not determined. In general, there is limited variation in timing of hatch (average hatch day over all years (±*SD*) = 32 ± 4 June days) and, therefore, we did not expect a marked difference in including season as June day or days until hatch for the subset of individuals for which hatch dates were known. This expectation was confirmed when we compared the outcome of two sets of analyses for the subset of females with a known hatching date (*N* = 1,082 FID observations, *N* = 212 females), including, respectively, days until hatch in the first models (see Table S1 for results) or June day in the second models (see Table S2 for results). We found no difference in the outcome of the first and second models and the parameter estimates were virtually similar. We therefore decided that modelling the seasonal component by using June day was justified and report on this in the results.

Second, we analysed if FID varied as a function of age by fitting age and age^2^ as fixed effects. The relationship between age and FID could be simply linear if females invest increasingly in reproduction over their lifetime (i.e. terminal investment: Clutton‐Brock, 1984). However, we also included age^2^ in the models because in long‐lived species the relationship between age and FID is expected to follow a U‐pattern (Møller & Nielsen, 2014; Ortega et al., 2017). Hence, we expected that risk‐taking would first increase with age and then gradually decline to values associated with lower fitness.

Behavioural habituation through repeated nest visits represents a general issue with repeated behavioural testing (see e.g. Araya‐Ajoy & Dingemanse, 2017; Class & Brommer, 2016; Knight & Temple, 1986). In this study, we dealt with habituation by fitting the number of visits to a female's nest across all study years as a fixed effect in all our analyses with 0 being the first visit to an individuals’ nest in the entire dataset, 1 the second visit etc. This method is deemed efficient for individuals that are visited several times per year (sensu Class & Brommer, 2016). However, because habituation and seasonal or age effects are inherently correlated (Figures S1 and S2), we performed additional analyses to better judge how estimates of plasticity in nest defence with season and age could be affected by habituation. To that end, we analysed variation in FID using models fitted with either June day or age as explanatory variables and compared them with models fitted with (a) repeated nest visits within years and June day (Table S3) and (b) repeated nest visits across years and age (Table S4) respectively.

ID, ID‐year and observer were fitted in all models as random effects. All variables were *z*‐transformed to a mean of zero and a standard deviation of one to improve interpretability of model estimates (Schielzeth & Forstmeier, 2009).

#### 2.4.3 Unravelling the underlying mechanisms of state dependence

To determine whether the covariation between FID and state (date or age) was caused by plasticity or selection (Figure 1), we used a within‐individual centring technique to separate within‐individual effects (involving phenotypic plasticity), from between‐individual effects (involving selection) of state on behaviour (de Pol & Wright, 2009; van Dingemanse et al., 2010). Within‐individual centring is necessary when not all individuals experience identical conditions for any within‐individual fixed effect; this happens for instance when individuals are not sampled over exactly the same range of the environmental gradient (here date and age; Araya‐Ajoy et al., 2015; Dingemanse & Dochtermann, 2013). We therefore calculated, for all females, the between‐individual variation component which was the individuals' mean June day/age (‘Mean’), and the within‐individual variation component by subtracting the individuals' mean June day/age from each observation value (‘Diff’; van de Pol & Wright, 2009). Models #2 thus included Mean June day/Mean Age, Diff June day/Diff Age and the number of nest visits as fixed effects and random intercepts for ID, ID‐year and observer. We found within‐individual effects (see Section 3) and therefore the logical next step was to examine whether there was significant between‐individual variation in the slopes of the within‐individual effect. Therefore, in the third models (#3) we further quantified between‐individual variation in slopes over the environmental gradient and compared these with models #2 using likelihood ratio tests (LRT). Next, we tested if intercepts and slopes were correlated. Significance of such a correlation was assessed using likelihood ratio tests between models that estimated or did not estimate covariance between intercepts and slopes (van de Pol, 2012).

For all models, we give model estimates of fixed (β) and random effects (variance) with their 95% credible intervals. For this, we used the ‘sim’ function of the package arm to simulate posterior distributions of the model parameters based on a 1,000 simulations (Gelman et al., 2018). 95% credible intervals (CIs) around the estimate were then extracted by calculating the highest posterior density intervals (Hadfield, 2010). We assessed the statistical significance of fixed effects on the basis of the 95% CIs. We regard a fixed effect to be significant in the frequentist's sense when the 95% CI does not overlap with 0 (see Nicolaus et al., 2016).

## 3 RESULTS

### 3.1 Long‐ and short‐term repeatability of female FID

Variance estimates were 1.17 for ID, 0.43 for ID‐year and 0.76 for residuals. Thus, females were consistent in their FID both on the short‐ (i.e. within‐individual‐between‐year: *r* = 0.71, CI = 0.69, 0.73) and long term (i.e. between‐individual: *r* = 0.55, CI = 0.53, 0.58).

### 3.2 FID and season

**FIGURE 2:**
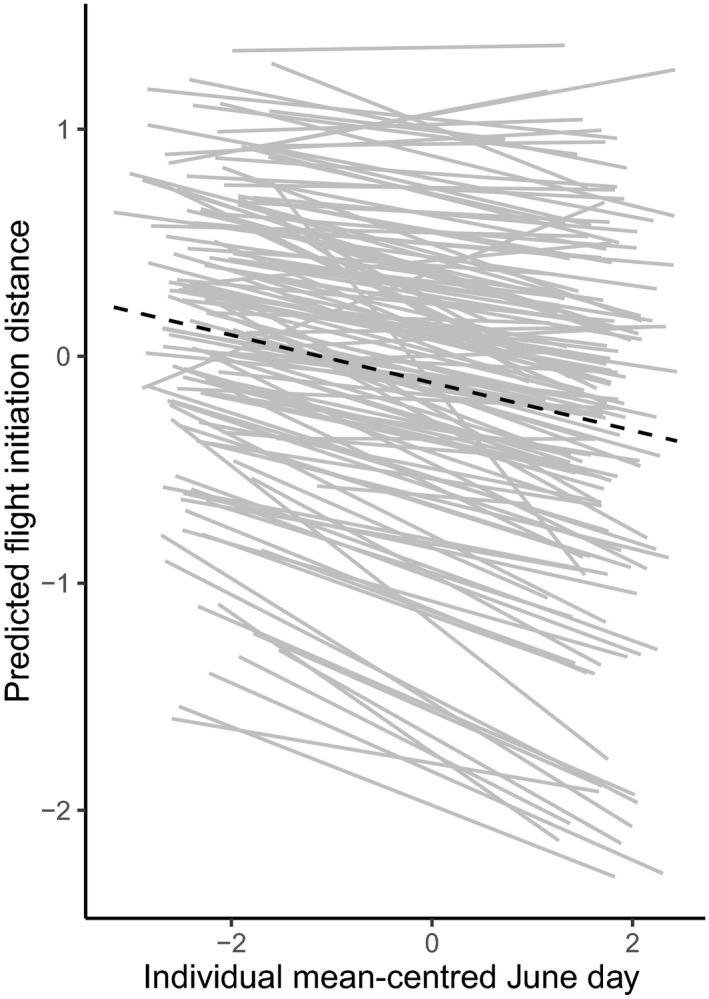
Predicted individual mean values of flight initiation distance (FID) as a function of individual mean‐centred June day. The grey lines represent a subset of 143 individuals with more than 10 measurements of FID (within‐individual response, model #3). The black dotted line represents population level seasonal response (model #1)

As predicted, FID decreased over the season, which indicated that female barnacle geese stayed on average longer on their nest upon approach later in the season (model #1, Table 1). Decomposing the seasonal effect into within‐ and between‐individual effects revealed that the population decline in FID over the season was mostly driven by individual plasticity in FID (significant effect of ‘Diff June day’), and not by selection (‘mean June day’ was not significant; model#2, Table 1). Further analyses revealed that females varied in their plastic response to date (I × E; LRT between model #2 and #3: *χ* ^2^ = 22.41, *df* = 2, *p* < 0.001). The model that allowed for a positive intercept‐slope covariance (model #3, Table 1) was preferred over the model without covariance (correlation_intercept‐slope_ = 0.66, LRT: *χ* ^2^ = 768.86, *df* = 2, *p* < 0.001), indicating that ‘bolder’ females (with a lower mean FID) exhibited stronger degree of behavioural plasticity compared to ‘shyer’ females (‘fanning out’ pattern; Figure 2).

**TABLE 1:** Model summary of three linear mixed‐effects models investigating variation in flight initiation distance as a function of time of the season (June day). Model #1 was used to investigate the overall population trend, model #2 was used to separate within‐individual effects (‘diff June day’) from between‐individual effects (‘mean June day’) and model #3 was used to investigate whether there was between‐individual variation in the slopes of the within‐individual effect (I × E). The included random effect ID represents individual identity and ID‐year the breeding attempt identity. (A) For each model, the predictions of the random regression variance components are given with the 95% credible intervals (CIs) in parentheses and (B) the estimates of the fixed effects are given with the 95% CIs in parentheses. Significant fixed effects are highlighted in bold

Season (A)					
Model	Random regression variance				
	ID	ID‐year	Observer	Residuals	I × E
#1	0.66 (0.59, 0.73)	0.14 (0.13, 0.15)	0.07 (0.05, 0.09)	0.32 (0.30, 0.33)	
#2	0.66 (0.60, 0.72)	0.14 (0.13, 0.15)	0.06 (0.05, 0.09)	0.32 (0.30, 0.33)	
#3		0.15 (0.14, 0.16)	0.08 (0.05, 0.11)	0.31 (0.30, 0.32)	0.02 (0.003, 0.70)

Season (B)					
Model	Fixed effects				
	Intercept	# Visits	June day	Mean June day	Diff June day
#1	−0.01 (−0.13, 0.18)	**−0.29 (−0.32, −0.24)**	**−0.07 (−0.09, −0.05)**		
#2	−0.03 (−0.14, 0.18)	**−0.28 (−0.32, −0.24)**		0.04 (−0.02, 0.09)	**−0.06 (−0.09, −0.04)**
#3	0.06 (−0.12, 0.19)	**−0.29 (−0.32, −0.24)**		0.04 (−0.01, 0.10)	**−0.06 (−0.08, −0.03)**

### 3.3 FID and age

**FIGURE 3:**
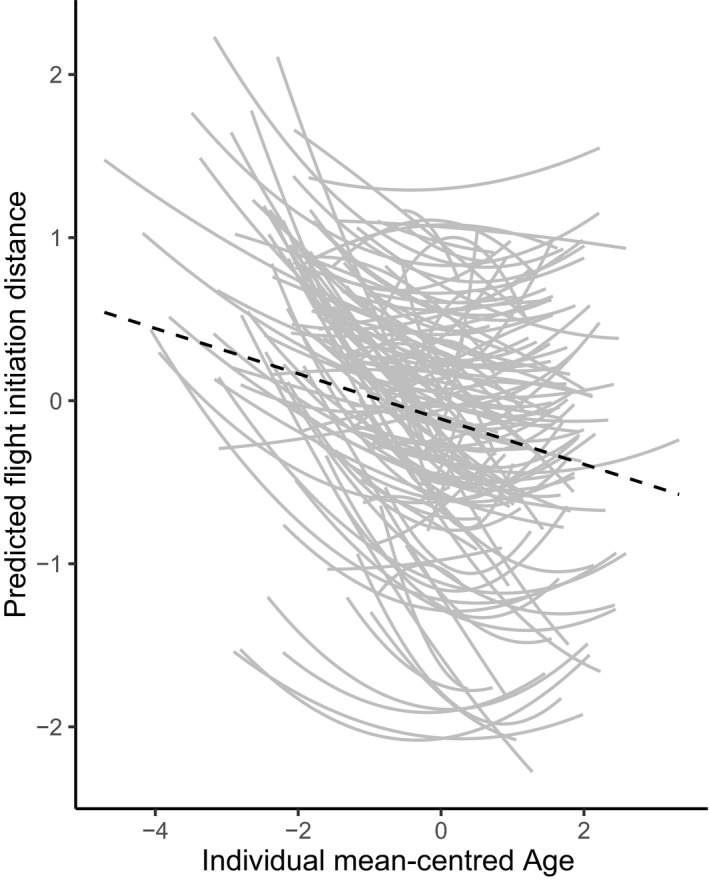
Predicted individual mean values of flight initiation distance (FID) as a function of individual mean‐centred age. The grey lines represent a subset of 134 individuals with a known age and with more than 10 measurements of FID in total (within‐individual effect, model #3). The black dotted line represents population level response (model #1)

Supporting our expectation, FID also declined significantly with age: younger females were on average shyer than older females (model #1, Table 2). We did not find a quadratic effect of age on population mean FID. The population decline in FID with age was due to quadratic individual plastic adjustment of FID with age (within‐individual effects; ‘Diff Age’) rather than selective (dis)appearance of shy females (non‐significant between‐individual effect ‘mean Age’: model #2, Table 2). Females differed significantly in their plastic adjustment of FID to age (significant I × E, LRT between model #2 and #3: *χ* ^2^ = 45.75, *df* = 2, *p* < 0.001) and plastic response was stronger for bolder females (correlation_intercept‐slope_ = 0.20, LRT: *χ* ^2^ = 803.43, *df* = 2, *p* < 0.001; ‘fanning out’ pattern; Figure 3, model #3 in Table 1).

**TABLE 2:** Model summary of three linear mixed‐effects models investigating variation in flight initiation distance as a function of individual age. Model #1 was used to investigate the overall population trend, model #2 was used to separate within‐individual effects (‘diff Age/diff Age^2^’) from between‐individual effects (‘mean Age/mean Age^2^’) and model #3 was used to investigate whether there was between‐individual variation in the slopes of the within‐individual effect (I × E). The included random effect ‘ID’ represents individual identity and ‘ID‐year’ the breeding attempt identity. (A) For each model, the predictions of the random regression variance components are given with the 95% credible intervals (CIs) in parentheses and (B) the estimates of the fixed effects are given with the 95% CIs in parentheses. Significant fixed effects are highlighted in bold

Age (A)					
Model	Random regression variance				
	ID	ID‐year	Observer	Residuals	I × E
#1	0.72 (0.62, 0.77)	0.13 (0.12, 0.14)	0.08 (0.06, 0.10)	0.32 (0.30, 0.33)	
#2	0.73 (0.63, 0.78)	0.12 (0.11, 0.13)	0.08 (0.06, 0.10)	0.32 (0.30, 0.33)	
#3		0.09 (0.08, 0.10)	0.08 (0.05, 0.09)	0.32 (0.30, 0.33)	0.03 (0.02, 0.81)

Age (B)								
Model	Fixed effects							
	Intercept	# Visits	Age	Age^2^	Mean Age	Mean Age^2^	Diff Age	Diff Age^2^
#1	−0.04 (−0.17, 0.15)	**−0.21 (−0.30, −0.15)**	**−0.18 (−0.33, −0.04)**	0.09 (−0.05, 0.21)				
#2	0.06 (−0.13, 0.21)	**−0.20 (−0.28, −0.09)**			0.17 (−0.07, 0.59)	−0.26 (−0.64, 0.04)	**−0.09 (−0.16, −0.02)**	**0.06 (0.04, 0.10)**
#3	0.01 (−0.14, 0.17)	**−0.30 (−0.39, −0.18)**			0.23 (−0.05, 0.57)	−0.24 (−0.61, 0.05)	−0.01 (−0.10, 0.07)	**0.10 (0.06, 0.13)**

### 3.4 Habituation effect

The total number of visits to nests of female geese was negatively correlated with FID in all models, suggesting that over time females became less sensitive to human disturbance (they stayed longer on their nest; Tables 1 and 2). The additional analyses revealed that plasticity of FID over the season occurred independently of habituation (Table S3) while plasticity of FID over age was in fact confounded with habituation (‘Diff Age’ became non‐significant in model #2, Table S4). In this latter case, controlling for habituation further revealed that individuals with longer FIDs lived longer (‘mean Age’ became significant in model #2, Table S4).

## 4 DISCUSSION

This study tested whether nest defence behaviour (flight initiation distance or FID; a measure of risk‐taking) was tuned to variation in individual ‘state’ (future fitness expectations) in free‐living female barnacle geese. We predicted that FID would decline as a function of time of the season and individual age, two state variables that can vary within and between females and reduce future fitness expectations. We detected that female FID was strongly repeatable at both the long term (i.e. between‐individual: 0.55) and short term (i.e. within‐individual‐between‐year: 0.71). As predicted, FID decreased over the season and over age for all females, showing that on average, females were bolder later in the season and at older age. Decomposing these population patterns into within‐ and between‐individual effects revealed that the declines in population mean FID over the season and over age were driven by individual plasticity in FID and not by selection. Females exhibited significant variation in plastic response (I × E) with bolder females being more responsive than shyer individuals (positive correlation between intercepts and slopes of the reaction norms). Below we discuss the ecological and evolutionary implications of our findings.

### 4.1 Nest defence behaviour as a personality trait

Our finding of high long‐ and short‐term repeatability of female FID is very similar to those found in a recent study on nest defence behaviour against a human intruder in a related goose species, the Canada goose *Branta canadensis* (Clermont et al., 2019: long‐term repeatability: 0.50, short‐term repeatability: 0.72). As found in previous studies (e.g. Kontiainen et al., 2009; Patrick et al., 2013), repeatability of nest defence appears to be relatively high for a behavioural trait (on average 0.37; Bell et al., 2009). Our study thereby adds to the evidence that individuals consistently differ in their tendency to take risks in protecting their offspring (e.g. Betini & Norris, 2012; Fresneau et al., 2014; Kontiainen et al., 2009; Patrick et al., 2013; Thys et al., 2019) despite exhibiting plasticity in that trait: that is, the relative rank of individuals is preserved along the environmental gradients. The substantial variation in the elevations of the FID behavioural reaction norms can originate from both from additive genetic variance and other, non‐heritable, stable differences between individuals, that is, ‘permanent environment effects’ (e.g. Dingemanse et al., 2010). Future studies should identify sources of variation in FID and establish if variation in barnacle goose nest defence is linked to fitness differences. This knowledge would be valuable, because if nest defence is linked to fitness, and is heritable, then it would have the potential to evolve under selection (Dingemanse & Reale, 2005).

### 4.2 Plastic adjustment of nest defence behaviour to changes in asset

Female barnacle geese plastically increased their nest defence over the season, as reflected by a decrease in their FID. Parental investment theory and the renesting potential hypothesis are two non‐exclusive explanations for this pattern (see Introduction; Shew et al., 2016). Arctic‐breeding birds such as barnacle geese experience a very short breeding season, as the snow‐free season is limited (mean snow‐free season length in Kongsfjorden is 81 days ± 12: Lameris et al., 2019) which seems to prohibit renesting (from laying till fledge takes approx. 71 days; see Methods). Renesting has rarely been observed in the Arctic and only seems to happen when clutches are depredated by polar bears *Ursus maritimus* early in the season when geese are still laying or have just started to incubate (J. Prop, pers. comm.; Mitchell et al., 1988). Hence, we consider it likely that females breeding in the Arctic adjust their nest defence to a decrease in asset over the season.

Supporting the asset protection principle (Clark, 1994), we also found that females increased their nest defence with age, that is, with decreasing asset (or decreasing future fitness expectations). We further detected a negative quadratic relationship between FID and age, meaning that nest defence first increased with age, reaching a high point with middle age and then declined with older age. Such age‐specific patterns that are bell‐ or inverted‐U‐shaped have been detected more often for reproductive rates, survival probabilities (e.g. Berman et al., 2009; Forslund & Pärt, 1995; Jones et al., 2008; Patrick & Weimerskirch, 2014; Rockwell et al., 1993) and, more recently, for offspring defence (Møller & Nielsen, 2014; Ortega et al., 2017). A possible explanation for our results is that, with increasing age, defence first increased with decreasing asset and improvements of competence (e.g. breeding experience, access to resources; Forslund & Pärt, 1995) and then gradually declined as a result of reduced reproductive performance (Rockwell et al., 1993).

The additional analyses on habituation revealed that the effect of season was independent of habituation (Table S3), but that the plastic effect of age on FID needs to be interpreted with more care. The within‐individual effect of age on FID appeared indeed to be confounded with habituation (Table S4; Figure S2), but controlling for habituation revealed a significant and positive between‐individual effect in this model which indicates selective disappearance of bold individuals (Table S4). These results imply that disregarding habituation can mask selective disappearance and cause an overestimated individual plasticity coefficient. Hence, future studies should include habituation effects.

### 4.3 Female differences in plasticity

Interestingly, our study revealed the existence of individual differences in plasticity with bolder females being generally more plastic than shyer individuals. Such differences in plasticity or reactivity between behavioural types have been extensively reported in the ‘coping style’ literature (Benus et al., 1987; Coppens et al., 2010; Koolhaas et al., 2010). However, in contrast to our findings, bolder individuals were often found to be less responsive to environmental change and assumed to rely mainly on internal routines (‘pro‐active’ coping style), while shyer individuals were more responsive to environmental change (‘re‐active’ coping style; e.g. Cornwell et al., 2019; Jolles et al., 2019; Kareklas et al., 2016; Koolhaas et al., 2010). The discrepancy found with the coping‐style literature supports a recent review showing that the relationships between personality and plasticity are often equivocal and lack consistency (Stamps, 2016).

Relatively few other studies have used a behavioural reaction norm approach to test for I × E in parental behaviours in general (Royle et al., 2014; Westneat et al., 2011) or, in nest defence behaviours in particular (Betini & Norris, 2012; Kontiainen et al., 2009; Thys et al., 2019). In line with our results, both in tree swallows *Tachycineta bicolor* (Betini & Norris, 2012) and Ural owls *Strix uralensis* (Kontiainen et al., 2009), bolder birds were found to be more plastic. In female great tits *Parus major*, however, no evidence was found for individual differences in plasticity (Thys et al., 2019).

Adaptive hypotheses for individual differences in plasticity are still under development (Stamps & Biro, 2016). Presently, we can only speculate about the causes and consequences of the observed individual differences in plasticity in FID of female barnacle geese (I × E). If the individual differences in plasticity are not caused by permanent environmental effects (e.g. maternal and natal effects) and are mirrored on the genetic level by genotype by environment (G × E) or by genotype by age (G × A) interactions, then this would imply that plasticity could evolve under selection (Brommer, 2013; Brommer & Class, 2015; Nussey et al., 2007). Furthermore, even though there may be a heritable basis to individual differences in plasticity, evolution can still be constrained because mean trait level and plasticity can be genetically correlated (Lande, 1979; Lande & Arnold, 1983). In our study, bolder females were more plastic. If this correlation translates at the genetic level, this means plasticity and boldness cannot evolve independently which may affect the trajectories and rates of evolutionary changes available to populations (Brommer, 2013).

Our study solely focused on females and it is currently unknown how nest defence behaviour varies in males, who also defend the nest. To unravel the exact adaptive mechanisms underlying the maintenance of variation in personality and plasticity, future studies on geese should investigate the realized fecundity and longevity of different behavioural types in both males and females.

## 5 CONCLUSIONS

In line with adaptive personality theory, we found that nest defence of barnacle goose females differs consistently among individuals and is adjusted to individual state (season and age). Additionally, this study revealed that behavioural types differ in their level of plasticity, with bolder females being generally more plastic than shyer females. We thus show that variation in state can explain the emergence of variation in behaviour. The exact mechanism needs further scrutinizing and future studies should focus on the persistence/enhancement of such variation which requires studying feedback loops between state and behaviour. Furthermore, it would be worthwhile quantifying the direct fitness consequences of individual variation in nest defence to formally establish the adaptive nature of variation in personality, plasticity and their correlation.

## AUTHORS' CONTRIBUTIONS

M.E.d.J. and M.J.J.E.L. conceived the ideas and designed methodology; M.E.d.J., M.J.J.E.L. and R.W.F. collected data; M.E.d.J. analysed the data with the help of M.N.; M.E.d.J. led the writing of the manuscript; M.E.d.J., M.N. and R.W.F. contributed critically to the drafts and all authors gave final approval for publication.

## Supporting information

Supplementary Material

## Data Availability

Data available from the Dryad Digital Repository https://doi.org/10.5061/dryad.sf7m0cg50 (de Jong et al., 2020).
