# Supplementary material for: State dependence explains individual variation in nest defence behaviour in a long‐lived bird
Source: J Anim Ecol. 2021 Jan 8;90(4):809–19. doi: 10.1111/1365-2656.13411 (PMC8048547; doi:10.1111/1365-2656.13411)
Supplement: Supplementary file 1 — Supplementary Material [file JANE-90-809-s001.docx]

**Supporting information**

**Table S1:** Summary of linear mixed-effects models investigating variation in flight initiation distance as a function of nest stage (i.e. number of days until hatch). For these analyses, we used the subset of females with a known hatching date (N = 1082 FID observations, N = 212 females). For both models, the estimates are given with the 95% credible intervals in parentheses of (A) the random regression variance components and (B) the fixed effects. Model #2 is preferred over Model #3 (χ^2^ = 1.45, df = 2, P = 0.485), and therefore the results of model #3 are not shown. Significant fixed effects are highlighted in bold.

| Nest stage  (A) | Model | Random regression variance | |  |  |
| --- | --- | --- | --- | --- | --- |
|  |  | ID | ID-year | Observer | Residuals |
|  | #1 | 0.63 (0.70, 0.96) | 0.08 (0.06, 0.08) | 0.04 (0.01, 0.07) | 0.29 (0.26, 0.32) |
|  | #2 | 0.60 (0.72, 0.96) | 0.08 (0.05, 0.08) | 0.04 (0.01, 0.07) | 0.29 (0.27, 0.32) |

| Nest stage  (B) | Model | Fixed effects | |  |  |  |
| --- | --- | --- | --- | --- | --- | --- |
|  |  | Intercept | # Visits | # Days until hatch | mean # Days until hatch | diff # Days until hatch |
|  | #1 | -0.05  (-2.66, 0.13) | **-0.18**  **(-0.28, -0.09)** | **0.11**  **(0.08, 0.15)** |  |  |
|  | #2 | -0.06  (-0.16, 0.13) | **-0.16**  **(-0.25, -0.06)** |  | **-0.12**  **(-0.21, -0.03)** | **0.11**  **(0.08, 0.16)** |

**Table S2:** Model summary of linear mixed-effects models investigating variation in flight initiation distance as a function of season (i.e. June day). For these analyses, we used the subset of females with a known hatching date (N = 1082 FID observations, N = 212 females). For both models, the estimates are given with the 95% credible intervals in parentheses of (A) the random regression variance components and (B) the estimates of the fixed effects ± standard error. Model #2 is preferred over Model #3 (χ^2^ = 0.69, df = 2, P = 0.709), and therefore the results of model #3 are not shown. Significant fixed effects are highlighted in bold.

| Season  (A) | Model | Random regression variance | |  |  |
| --- | --- | --- | --- | --- | --- |
|  |  | ID | ID-year | Observer | Residuals |
|  | #1 | 0.63 (0.67, 0.97) | 0.08 (0.05, 0.08) | 0.04 (0.02, 0.10) | 0.29 (0.27, 0.32) |
|  | #2 | 0.58 (0.62, 0.91) | 0.08 (0.06, 0.08) | 0.04 (0.02, 0.08) | 0.29 (0.26, 0.31) |

| Season  (B) | Model | Fixed effects | |  |  |  |
| --- | --- | --- | --- | --- | --- | --- |
|  |  | Intercept | # Visits | June day | mean June day | diff June day |
|  | #1 | -0.06  (-0.26, 0.11) | **-0.17**  **(-0.26, -0.07)** | **-0.11**  **(-0.15, -0.07)** |  |  |
|  | #2 | -0.04  (-0.26, 0.13) | **-0.18**  **(-0.28, -0.10)** |  | **0.16**  **(0.07, 0.26)** | **-0.11**  **(-0.14, -0.07)** |

**Table S3:** A comparison of two mixed-effects models to investigate whether estimates of plasticity to June day change when habituation within years is taken into account. Model #1 includes June day and model #2 includes June day and the number of repeats within years. For both models, the estimates are given with the 95% credible intervals in parentheses of (A) the estimates of the random regression variance components and (B) the fixed effects. Significant fixed effects are highlighted in bold.

| Season  (A) | Model | Random regression variance | |  |  |  |
| --- | --- | --- | --- | --- | --- | --- |
|  |  | ID-year | Observer | Residuals | I x E | Correlation I x E |
|  | #1 | 0.20 (0.19, 0.22) | 0.04 (0.02, 0.05) | 0.31 (0.30, 0.33) | 0.02 (0.002, 0.68) | 0.74 |
|  | #2 | 0.20 (0.18, 0.21) | 0.04 (0.02, 0.06) | 0.31 (0.30, 0.33) | 0.02 (0.002, 0.69) | 0.74 |

| Season  (B) | Model | Fixed effects | |  |  |  |
| --- | --- | --- | --- | --- | --- | --- |
|  |  | Intercept | mean June day | diff June day | mean Visit number within years | diff Visit number within years |
|  | #1 | 0.14  (0.04, 0.30) | 0.04  (-0.04, 0.08) | **-0.08**  **(-0.11, -0.06)** |  |  |
|  | #2 | 0.16  (0.01, 0.29) | -0.006  (-0.10, 0.09) | **-0.14**  **(-0.19, -0.10)** | 0.03  (-0.07, 0.13) | **0.06**  **(0.02, 0.11)** |

**Table S4:** A comparison of two mixed-effects models years to investigate whether estimates of plasticity to age change when habituation across years is taken into account. Model #1 includes individual age and model #2 includes age and the number of repeats across years. For each model, the estimates are given with the 95% credible intervals in parentheses of (A) the random regression variance components and (B) the fixed effects. No evidence was found for a quadratic effect of visit number across years, and therefore this is not shown. Significant fixed effects are highlighted in bold.

| Age  (A) | Model | Random regression variance | |  |  |  |
| --- | --- | --- | --- | --- | --- | --- |
|  |  | ID-year | Observer | Residuals | I x E | Correlation I x E |
|  | #1 | 0.09 (0.08, 0.09) | 0.08 (0.05, 0.10) | 0.32 (0.31, 0.33) | 0.02 (0.01, 0.85) | 0.19 |
|  | #2 | 0.09 (0.08, 0.10) | 0.07 (0.05, 0.09) | 0.33 (0.30, 0.33) | 0.03 (0.01, 0.83) | 0.21 |

| Age  (B) | Model | Fixed effects | |  |  |  |  |  |
| --- | --- | --- | --- | --- | --- | --- | --- | --- |
|  |  | Intercept | mean Age | mean Age^2^ | diff Age | diff Age^2^ | mean Visit number across years | diff Visit number across years |
|  | #1 | 0.07  (-0.07, 0.26) | 0.09  (-0.23, 0.36) | -0.20  (-0.48, 0.14) | **-0.21**  **(-0.24, -0.16)** | **0.06**  **(0.03, 0.10)** |  |  |
|  | #2 | -0.008  (-0.18, 0.14) | **0.31**  **(0.01, 0.62)** | **-0.25**  **(-0.66,**  **-0.03)** | -0.04  (-0.15, 0.05) | **0.09**  **(0.06, 0.13)** | **-0.23**  **(-0.34, -0.14)** | **-0.14**  **(-0.26, -0.06**) |


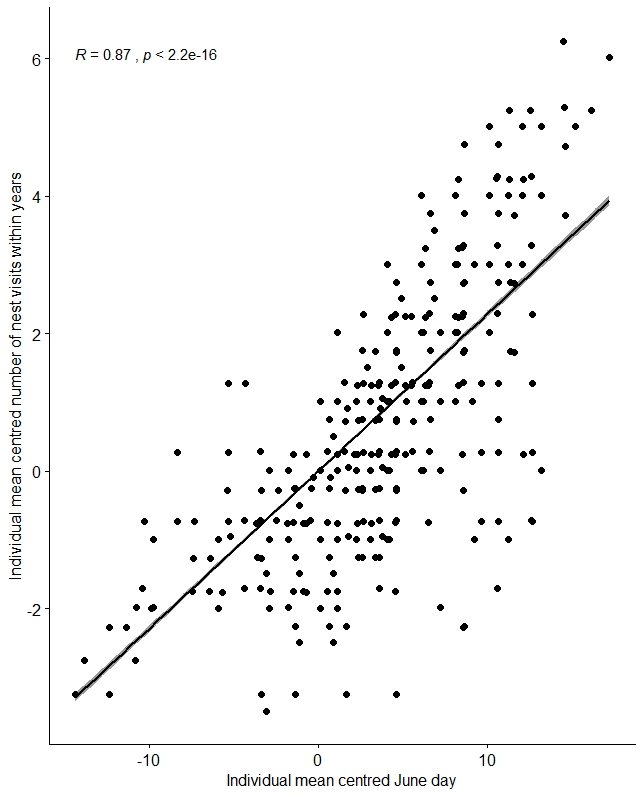


**Figure S1:** Correlation between individual mean centred June day and individual mean centred number of nest visits within years.


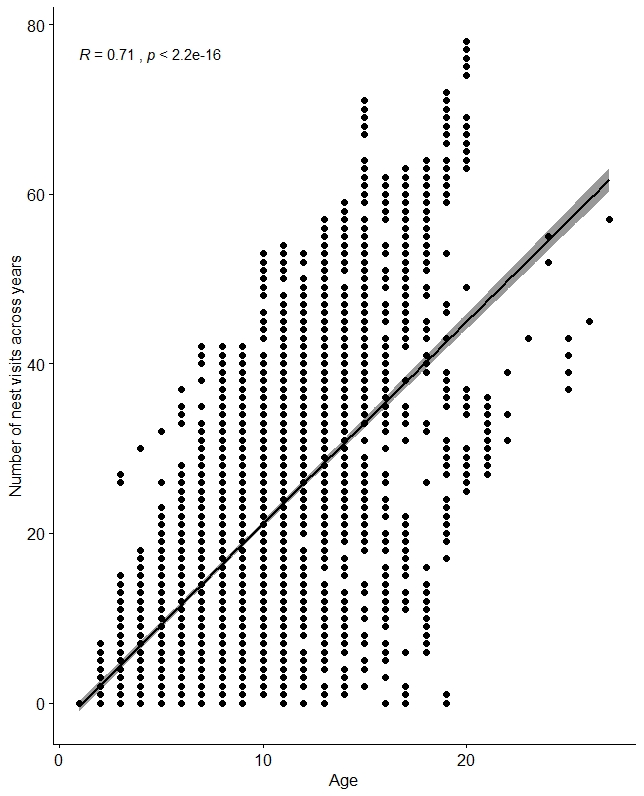


**Figure S2:** Correlation between individual age and the number of nest visits across years.
